# Supplementary material for: Destabilization of chromosome structure by histone H3 lysine 27 methylation
Source: PLoS Genet. 2019 Apr 22;15(4):e1008093. doi: 10.1371/journal.pgen.1008093 (PMC6510446; doi:10.1371/journal.pgen.1008093)
Supplement: S1 Supplementary Text — (PDF) [file pgen.1008093.s013.pdf]

# Destabilization of chromosome structure by histone H3 lysine 27 methylation

Mareike Möller[1,2], Klaas Schotanus[3], Jessica L. Soyer[4], Janine Haueisen[1,2], Kathrin Happ[1], Maja Stralucke[1], Petra Happel[5], Kristina M. Smith[6], Lanelle R. Connolly[7], Michael Freitag[7], Eva H. Stukenbrock[1,2*]

[1] Environmental Genomics, Christian-Albrechts University, Am Botanischen Garten 1-9, D-24118 Kiel, Germany

[2] Max Planck Fellow Group Environmental Genomics, Max Planck Institute for Evolutionary Biology, D-24306 Plön, Germany

[3] Department of Molecular Genetics and Microbiology, Duke University Medical Center, Durham, North Carolina, United States of America

[4] UMR BIOGER, INRA, AgroParisTech, Université Paris-Saclay, 78850 Thiverval-Grignon, France

[5] Max Planck Institute for Terrestrial Microbiology, Karl-von-Frisch-Straße 10, D-35043 Marburg, Germany

[6] Department of Biology, Oregon State University - Cascades, Bend, OR 97702, United States of America

[7] Department of Biochemistry and Biophysics, Oregon State University, Corvallis, OR 97331-7305, United States of America

**Supplementary text S1.** Data analysis – programs and commands used for analysis of ChIP-seq, RNA-seq and genome sequencing data.

## 1. ChIP-sequencing

### Quality filtering

FASTX Toolkit - version 0.0.14 (http://hannonlab.cshl.edu/fastx_toolkit/)

removal of remaining adapter sequences (if necessary)
***fastx_clipper*** *-C -M 40 -a adapter_sequence -v -i input.fastq -o output.fastq*

trim low quality bases (-f6 for 50 nt reads, -f8 for 150 nt reads samples)
***fastx_trimmer*** *-Q33 -f6/8 -v -i input.fastq -o output.fastq*

remove low quality reads
***fastq_quality_filter*** *-Q33 -v -q 20 -p 80 -i input.fastq -o output.fastq*

### Mapping

bowtie2 version 2.1.0 [1]

***bowtie2*** *-p 6 -x reference_genome -U quality_filtered_input.fastq -S output.sam*

### Converting to bam files and sorting

Samtools version 1.5 [2]

***samtools view*** *-@ 6 -Sb input.sam > output.bam*
***samtools sort*** *-@ 6 input.bam > output_sorted.bam*
***samtools index*** *input_sorted.bam*

### Visualization of data

deeptools version 2.5.3 [3]

data is normalized to 1x coverage
***bamCoverage*** *--bam input_sorted.bam -o output.bw -p 6 –normalizeTo1x 39686251 --ignoreForNormalization chr_18 --binSize 10 --extendReads 150*

**Peak calling**
Homer version 4.9 [4]

***makeTagDirectory*** *name_tag_directory -mapq 1 input.sam*

H3K4me2
***findPeaks*** *name_tag_directory -style histone -region -size 1000 -minDist 500 -C 0 > output_peaks.txt*

H3K9me3 and H3K27me3
***findPeaks*** *name_tag_directory -style histone -region -size 2000 -minDist 2000 -C 0 > output_peaks.txt*

convert .txt to .bed
***pos2bed.pl*** *input_peaks.txt > output_peaks.bed*

**ChIP peak analysis**

bedtools version 2.25.0 [5]

merge peaks of replicates, output is overlapping regions
***bedtools intersect*** *-a input_replicate1_peaks.bed -b input_replicate2_peaks.bed > output_peaks_intersect.bed*

compute genome coverage of identified peaks
***bedtools genomecov*** *-i input_peaks_intersect.bed -g reference.genome > output_intersect_genomecoverage.txt*

annotate overlap of peaks with genes or transposable elements
***bedtools annotate*** *-i genes/TEs.bed -files input_peaks_intersect.bed > input_peaks_intersect _overlap_genes/TEs.txt*

## 2. RNA-sequencing

### Quality Filtering

Trimmomatic version 0.33 [6]

keep only 150bp reads
***java -jar trimmomatic-0.33.jar*** *SE -phred33 input.fastq output.fastq MINLEN:150*

trim low quality bases
***java -jar trimmomatic-0.33.jar*** *SE -phred33 input.fastq output.fastq HEADCROP:15*

FASTX Toolkit version 0.0.14 (http://hannonlab.cshl.edu/fastx_toolkit/)

***fastq_quality_filter*** *-Q33 -v -q 20 -p 80 -i input.fastq -o output.fastq*

***fastq_masker*** *-q 20 -r N -v -Q33 -i input.fastq -o output.fastq*

### Mapping

hisat2 (version 2.0.3-beta) [7]

dataset is reverse-stranded, maximum intron length is set to 1500 bp
***hisat2*** *-p8 --rna-strandness R --max-intronlen 1500 -x reference_genome -U input_masked.fastq –S output.sam*

### Converting to bam files and sorting

Samtools version 1.5 [2]

***samtools view*** *-@ 6 -Sb input.sam > output.bam*
***samtools sort*** *-@ 6 input.bam > output_sorted.bam*
***samtools index*** *input_sorted.bam*

### Count reads on features

htseq version 0.6.0 [8]

count reads on genes using the sorted bam files
***htseq-count*** *–f bam –m union –type=gene --idattr=Name --stranded=reverse input_sorted.bam reference.gff > output.txt*

### Calculate RPKM values

cuffdiff version 2.1.1 [9]

***cuffdiff*** *-p 6 -L cond1,cond2,cond3,cond4 --library-norm-method geometric --dispersion-method per-condition reference.gff*
*cond1_rep1_sorted.bam,cond1_rep2_sorted.bam*
*cond2_rep1_sorted.bam,cond2_rep2_sorted.bam*
*cond3_rep1_sorted.bam,cond3_rep2_sorted.bam*
*cond4_rep1_sorted.bam,cond4_rep2_sorted.bam*

## 3. Genome sequencing

### Quality Filtering

Trimmomatic (version 0.36) [6]

***java -jar trimmomatic-0.36.jar*** *PE -threads 8 -phred33 input_R1.fastq.gz input_R2.fastq.gz output_R1_paired.fastq output_R1_unpaired.fastq output_R2_paired.fastq output_R2_unpaired.fastq HEADCROP:5 CROP:145 LEADING:3 TRAILING:3 SLIDINGWINDOW:4:15 MINLEN:50*

### Mapping

bowtie2 version 2.1.0 [1]

***bowtie2*** *-p 8 -x reference_genome -1 input_R1_paired.fastq -2 input_R2_paired.fastq -S output.sam*

### Converting to bam files and sorting

samtools version 1.5 [2]

***samtools view*** *-@ 6 -Sb input.sam > output.bam*
***samtools sort*** *-@ 6 input.bam > output_sorted.bam*
***samtools index*** *input_sorted.bam*

### Visualization of data

deeptools version 2.5.3 [3]

data is normalized to 1x coverage
***bamCoverage*** *--bam input_sorted.bam -o output.bw --binSize 10 --normalizeTo1x 39686251*

**Detecting structural variation**

speedseq version 0.1.2 [10]

***speedseq align*** *-t 6 -R "@RG\tID:id\tSM:sample\tLB:lib" *
*reference_genome.fasta *
*input_R1_paired.fastq *
*input_R2_paired.fastq*

lumpy version 0.2.13 [11]

***lumpyexpress*** *-B speedseq_output_paired.fastq.bam -S speedseq_output_paired.fastq.splitters.bam -D speedseq_output_paired.fastq.discordants.bam -o output_lumpy.vcf*

# References


1. Langmead B, Salzberg SL. Fast gapped-read alignment with Bowtie 2. Nat Methods. 2012;9: 357–359.
2. Li H. A statistical framework for SNP calling, mutation discovery, association mapping and population genetical parameter estimation from sequencing data. Bioinformatics. 2011;27: 2987–2993.
3. Ramírez F, Ryan DP, Grüning B, Bhardwaj V, Kilpert F, Richter AS, et al. deepTools2: a next generation web server for deep-sequencing data analysis. Nucleic Acids Res. 2016;44: W160–W165.
4. Heinz S, Benner C, Spann N, Bertolino E, Lin YC, Laslo P, et al. Simple Combinations of Lineage-Determining Transcription Factors Prime cis-Regulatory Elements Required for Macrophage and B Cell Identities. Mol Cell. Elsevier Inc.; 2010;38: 576–589.
5. Quinlan AR, Hall IM. BEDTools: a flexible suite of utilities for comparing genomic features. Bioinformatics. 2010;26: 841–842.
6. Bolger AM, Lohse M, Usadel B. Trimmomatic: A flexible trimmer for Illumina sequence data. Bioinformatics. 2014;30: 2114–2120.
7. Kim D, Langmead B, Salzberg SL. HISAT: a fast spliced aligner with low memory requirements. Nat Methods. 2015;12: 357–60.
8. Anders S, Pyl PT, Huber W. HTSeq-A Python framework to work with high-throughput sequencing data. Bioinformatics. 2015;31: 166–169.
9. Trapnell C, Williams BA, Pertea G, Mortazavi A, Kwan G, van Baren MJ, et al. Transcript assembly and quantification by RNA-Seq reveals unannotated transcripts and isoform switching during cell differentiation. Nat Biotechnol. 2010;28: 511–515.
10. Chiang C, Layer RM, Faust GG, Lindberg MR, Rose DB, Garrison EP, et al. SpeedSeq: Ultra-fast personal genome analysis and interpretation. Nat Methods. 2015;12: 966–968.
11. Layer RM, Chiang C, Quinlan AR, Hall IM. LUMPY: A probabilistic framework for structural variant discovery. Genome Biol. 2014;15: 1–19.
