# Supplementary material for: Destabilization of chromosome structure by histone H3 lysine 27 methylation
Source: PLoS Genet. 2019 Apr 22;15(4):e1008093. doi: 10.1371/journal.pgen.1008093 (PMC6510446; doi:10.1371/journal.pgen.1008093)
Supplement: S1 Fig — (PDF) [file pgen.1008093.s014.pdf]

***Zymoseptoria tritici* strains used in this study**
FigS2
TableS1

- Zt09 wild type
- Δ*kmt1* ~~H3K9me3~~
- Δ*kmt6* ~~H3K27me3~~
- Δ*k1/6* ~~H3K27me3~~ ~~H3K9me3~~

	**Phenotypic characterization**	**Chromatin analyses**	**Transcriptome analyses**	**Accessory chromosome stability analyses**	**Genome stability analyses**
**Key finding**	*Impaired growth and virulence of Δkmt1 and Δk1/6 strains*	*Redistribution of H3K27me3 in absence of H3K9me3*	*Expression of TEs in absence of H3K9me3; little impact on gene expression by removing H3K27me3*	*Absence of H3K27me3 stabilizes accessory chromosomes; loss of H3K9me3 increases loss rate*	*Absence of H3K9me3 and redistribution of H3K27me3 results in large genome wide rearrangements*
**Experiment**	*In vitro* and *in planta* assays	ChIP-seq	RNA-seq	Short-term evolution experiment<br>Chromosome loss PCR screening	Long-term evolution experiment<br>Genome sequencing
**Figures and Tables**	FigS4<br>FigS5<br>FigS6<br>FigS7	Fig1<br>Fig2<br>FigS3<br><br>Table 1<br>Table S2	Fig3<br><br>Table S2<br>Table S3<br>Table S4<br>Table S5<br>Table S6<br>Table S7<br>Table S8	FigS8<br><br>Table 2	Fig4<br>Fig5<br>FigS8<br>FigS9<br>FigS10<br><br>Table S9<br>Table S10<br>Table S11<br>Table S12
