# Supplementary material for: Destabilization of chromosome structure by histone H3 lysine 27 methylation
Source: PLoS Genet. 2019 Apr 22;15(4):e1008093. doi: 10.1371/journal.pgen.1008093 (PMC6510446; doi:10.1371/journal.pgen.1008093)
Supplement: S2 Fig — Southern blots to confirm correct integration of deletion and complementation constructs: for deletion of kmt1 (A), complementation of kmt1 (B), deletion of kmt1 in a kmt6 deletion background resulting in the generation of a double deletion mutant (C), deletion of kmt6 (D), and complementation of kmt6 (E). The left blot verifying the deletion of kmt6 (D) displays two bands instead of one for the wildtype Zt09. This is likely due to incomplete digestion, as a second blot (on the right) using the same enzymes and probes only results in the correct band for Zt09. Depictured are genomic locations of wildtype (Zt09) and mutant strains, restriction enzymes used, probes, and expected fragment sizes on the blots. All tested strains, except for the underlined, were verified as correct mutants. The strains used for experiments in this study are highlighted in bold. (PDF) [file pgen.1008093.s015.pdf]

A

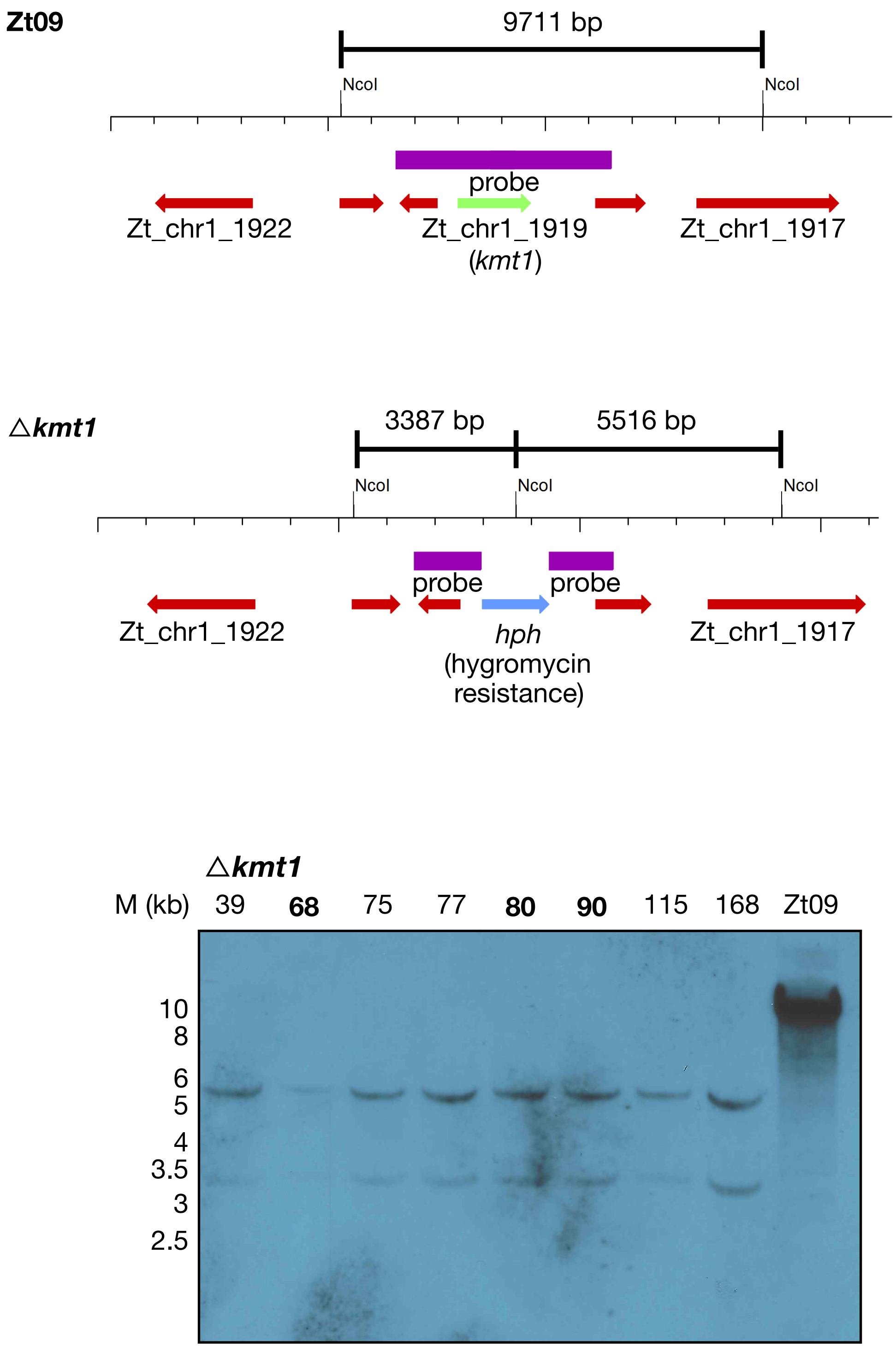
Zt09
9711 bp
NcoI
NcoI
probe
Zt_chr1_1922
Zt_chr1_1919
(kmt1)
Zt_chr1_1917
△kmt1
3387 bp
5516 bp
NcoI
NcoI
NcoI
probe
probe
Zt_chr1_1922
hph
(hygromycin
resistance)
Zt_chr1_1917
△kmt1
M (kb) 39 68 75 77 80 90 115 168 Zt09
10
8
6
5
4
3.5
3
2.5

B

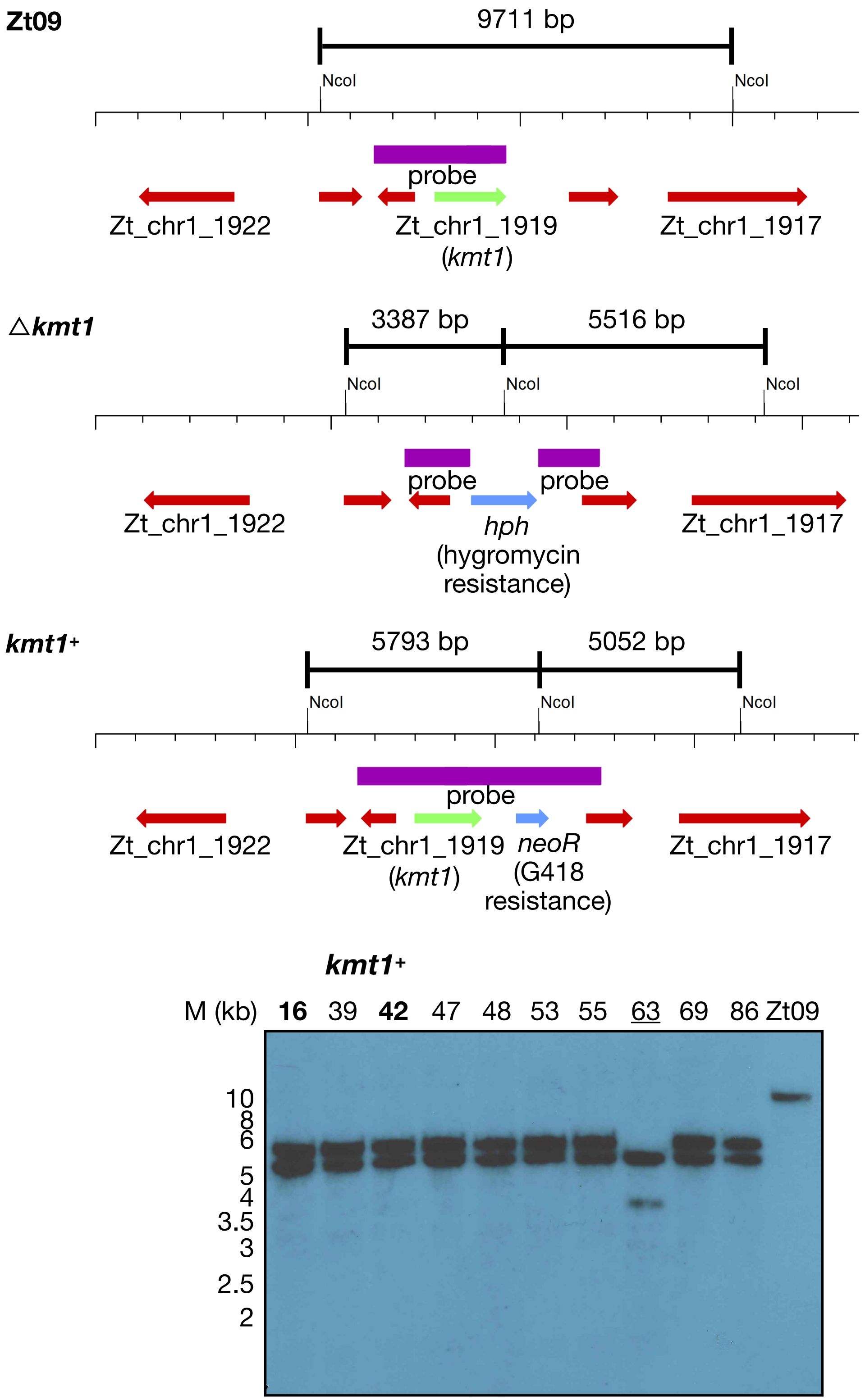

Zt09
9711 bp
NcoI
NcoI
probe
Zt_chr1_1922
Zt_chr1_1919
(kmt1)
Zt_chr1_1917
△kmt1
3387 bp
5516 bp
NcoI
NcoI
NcoI
probe
probe
Zt_chr1_1922
hph
(hygromycin
resistance)
Zt_chr1_1917
kmt1+
5793 bp
5052 bp
NcoI
NcoI
NcoI
probe
Zt_chr1_1922
Zt_chr1_1919
(kmt1)
neoR
(G418
resistance)
Zt_chr1_1917
kmt1+
M (kb) 16 39 42 47 48 53 55 63 69 86 Zt09
10
8
6
5
4
3.5
3
2.5
2

C

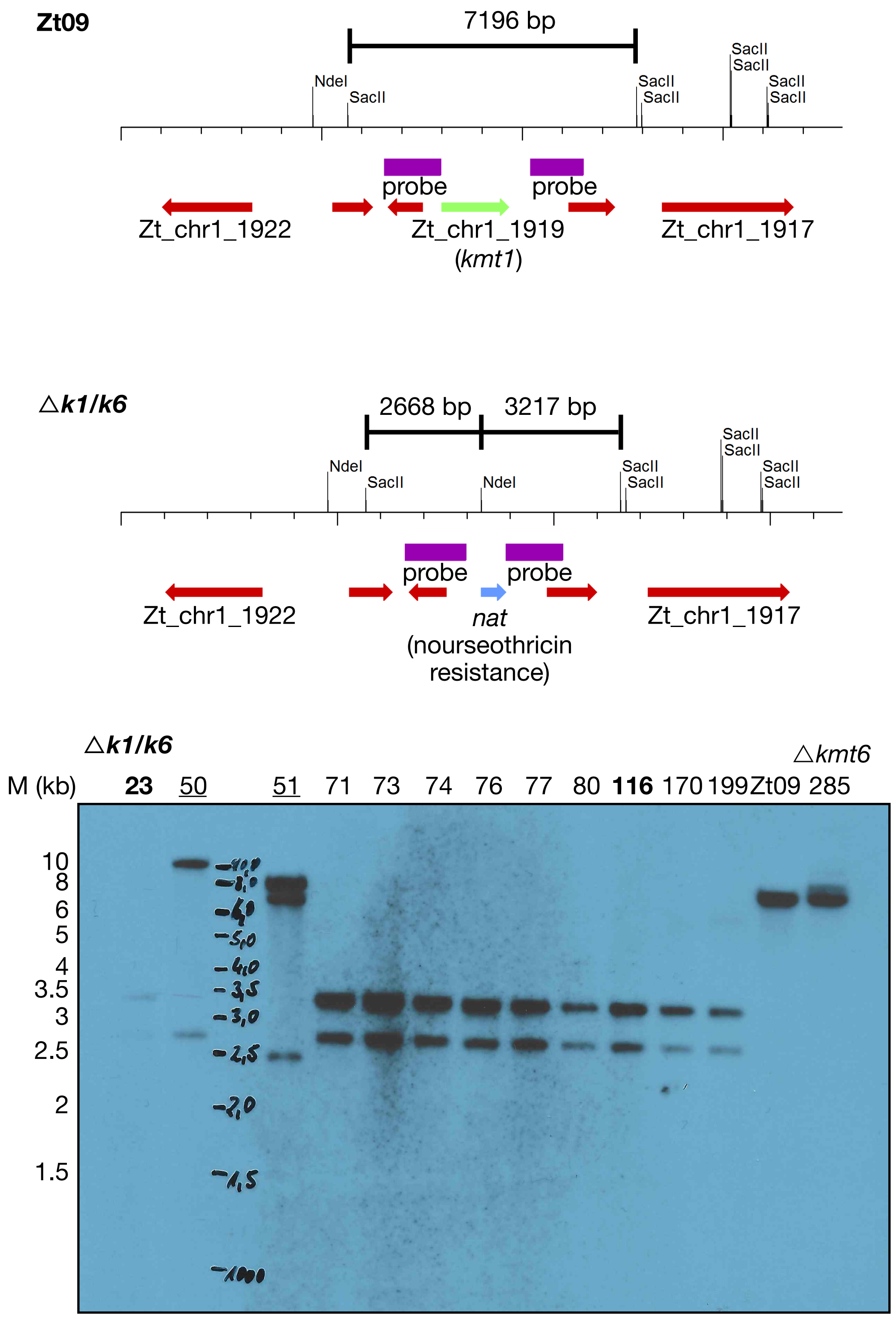
Zt09
7196 bp
NdeI
SacII
SacII
SacII
SacII
SacII
SacII
SacII
probe
probe
Zt_chr1_1922
Zt_chr1_1919
(kmt1)
Zt_chr1_1917
△k1/k6
2668 bp
3217 bp
NdeI
SacII
NdeI
SacII
SacII
SacII
SacII
SacII
SacII
probe
probe
Zt_chr1_1922
nat
(nourseothricin
resistance)
Zt_chr1_1917
△k1/k6
△kmt6
M (kb)
23
50
51
71
73
74
76
77
80
116
170
199
Zt09
285
10
8
6
5
4
3.5
3
2.5
2
1.5

D

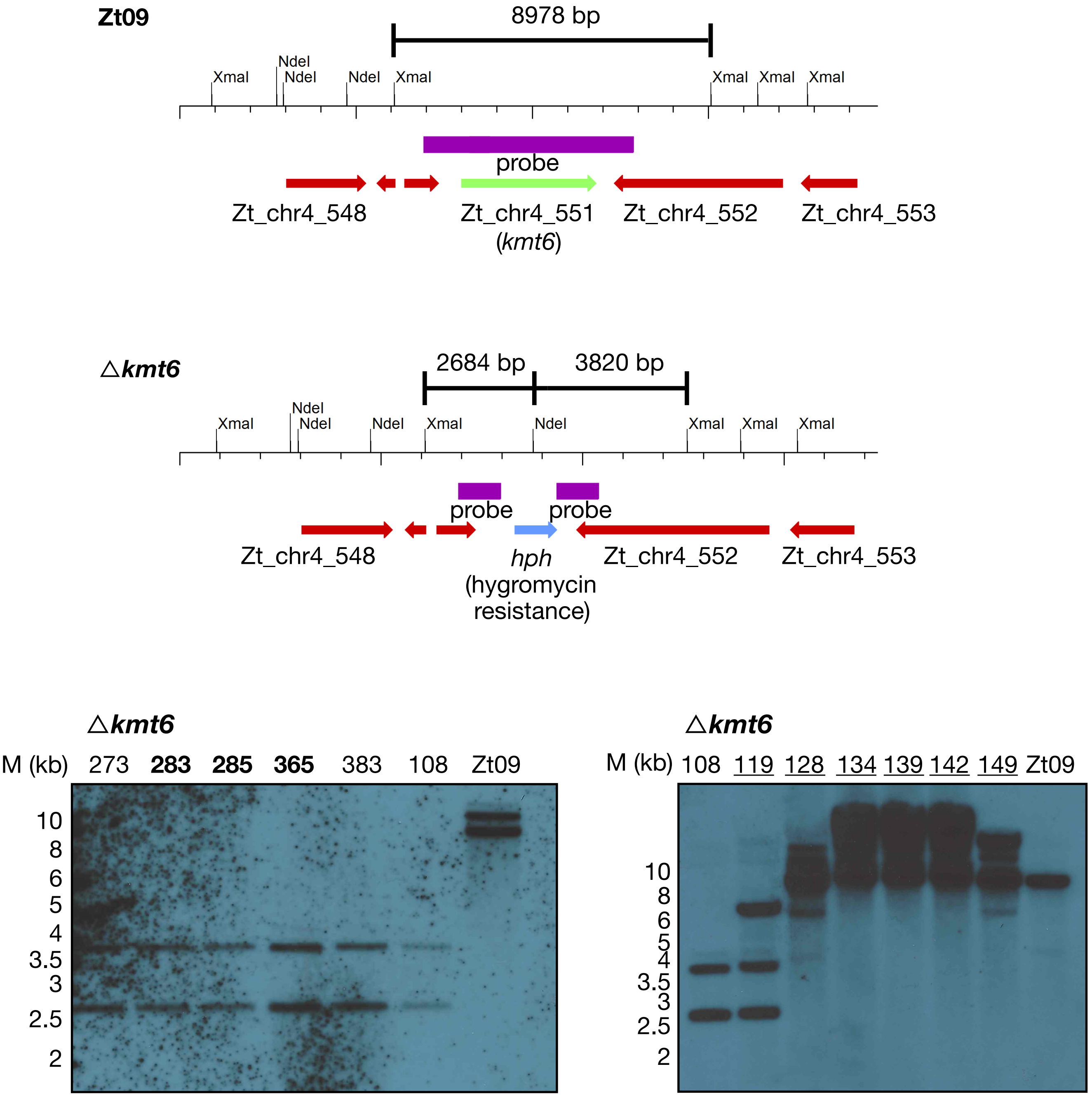
Zt09
8978 bp
XmaI
NdeI
NdeI
NdeI
XmaI
XmaI
XmaI
XmaI
probe
Zt_chr4_548
Zt_chr4_551
(kmt6)
Zt_chr4_552
Zt_chr4_553
△kmt6
2684 bp
3820 bp
XmaI
NdeI
NdeI
NdeI
XmaI
NdeI
XmaI
XmaI
XmaI
probe
probe
Zt_chr4_548
hph
(hygromycin
resistance)
Zt_chr4_552
Zt_chr4_553
△kmt6
M (kb) 273 283 285 365 383 108 Zt09
10
8
6
5
4
3.5
3
2.5
2
△kmt6
M (kb) 108 119 128 134 139 142 149 Zt09
10
8
6
5
4
3.5
3
2.5
2

E

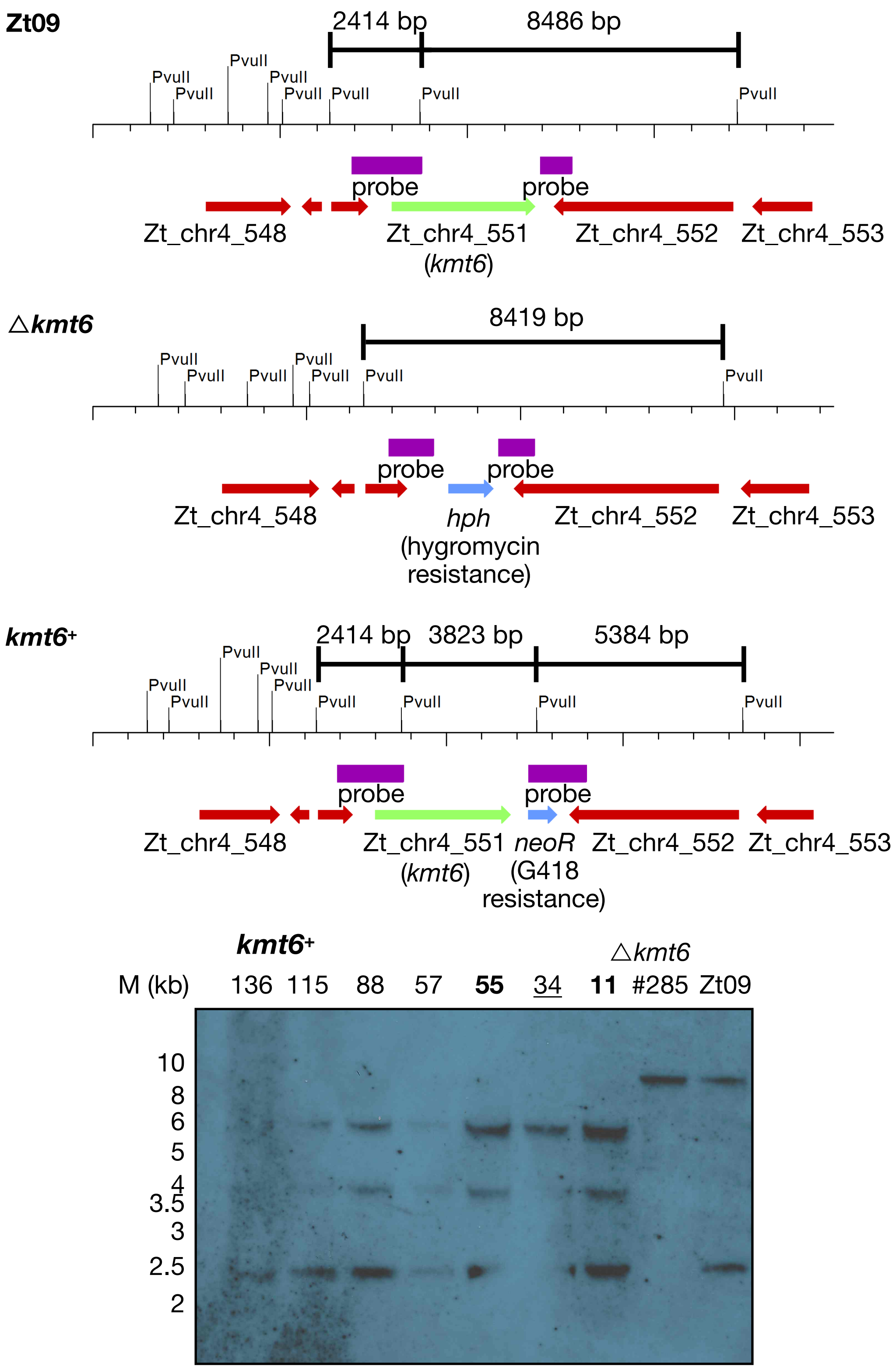
Zt09
2414 bp
8486 bp
PvuII
probe
probe
Zt_chr4_548
Zt_chr4_551
(kmt6)
Zt_chr4_552
Zt_chr4_553
△kmt6
8419 bp
PvuII
probe
probe
Zt_chr4_548
hph
(hygromycin
resistance)
Zt_chr4_552
Zt_chr4_553
kmt6+
2414 bp
3823 bp
5384 bp
PvuII
probe
probe
Zt_chr4_548
Zt_chr4_551
(kmt6)
neoR
(G418
resistance)
Zt_chr4_552
Zt_chr4_553
kmt6+
△kmt6
M (kb)
136
115
88
57
55
34
11
#285
Zt09
10
8
6
5
4
3.5
3
2.5
2
