# Supplementary figures and images for: Destabilization of chromosome structure by histone H3 lysine 27 methylation
Source: PLoS Genet. 2019 Apr 22;15(4):e1008093. doi: 10.1371/journal.pgen.1008093 (PMC6510446; doi:10.1371/journal.pgen.1008093)

**A**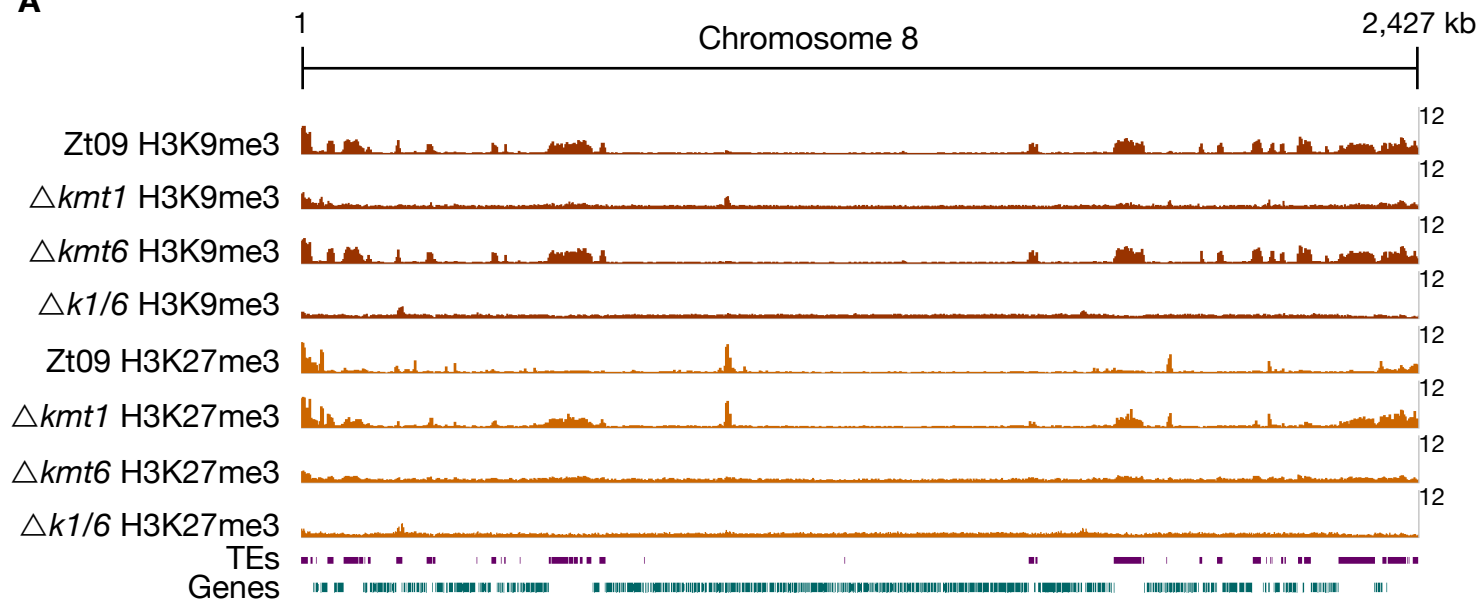**B**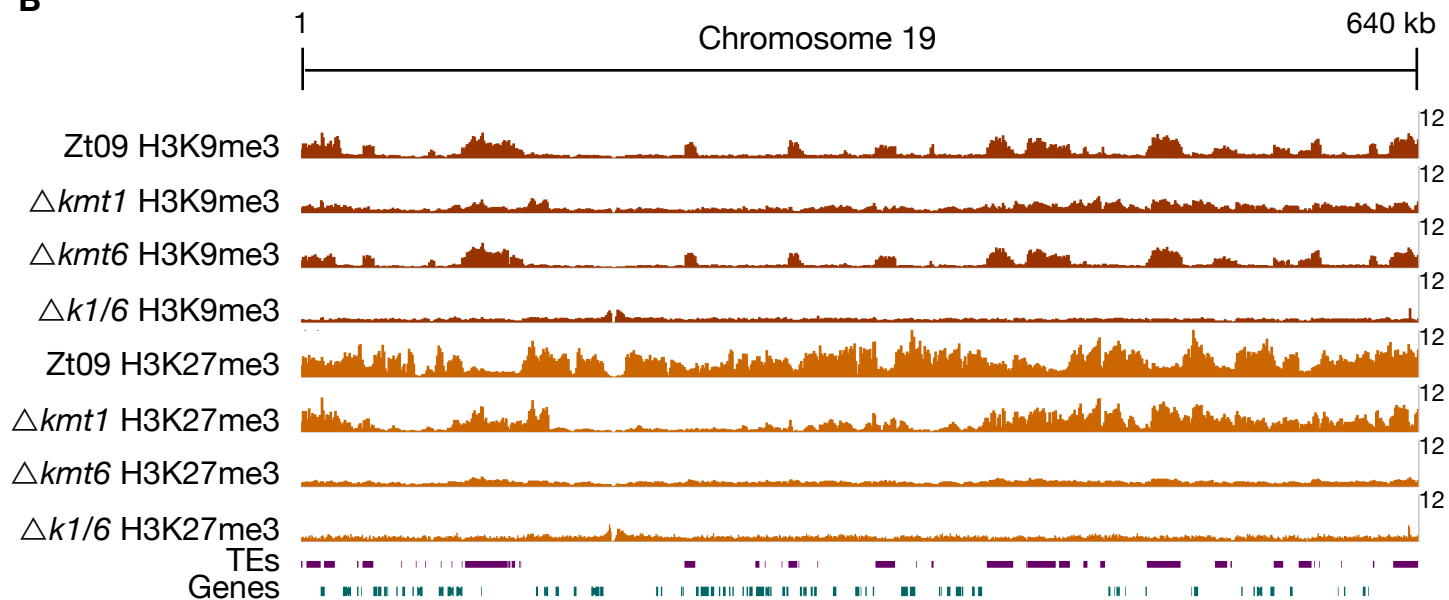

Supplement: S3 Fig — Shown are the ChIP-seq coverage tracks (normalized to 1x coverage with deeptools (115)) of one replicate per strain. As an example, the coverage of core chromosome 8 (A) and accessory chromosome 19 (B) is displayed. Based on the missing coverage, we confirm absence of H3K9me3 in the Δkmt1 and the Δk1/k6 strains and loss of H3K27me3 in the Δkmt6 and Δk1/k6 strains. (PDF) [file pgen.1008093.s016.pdf]

**A**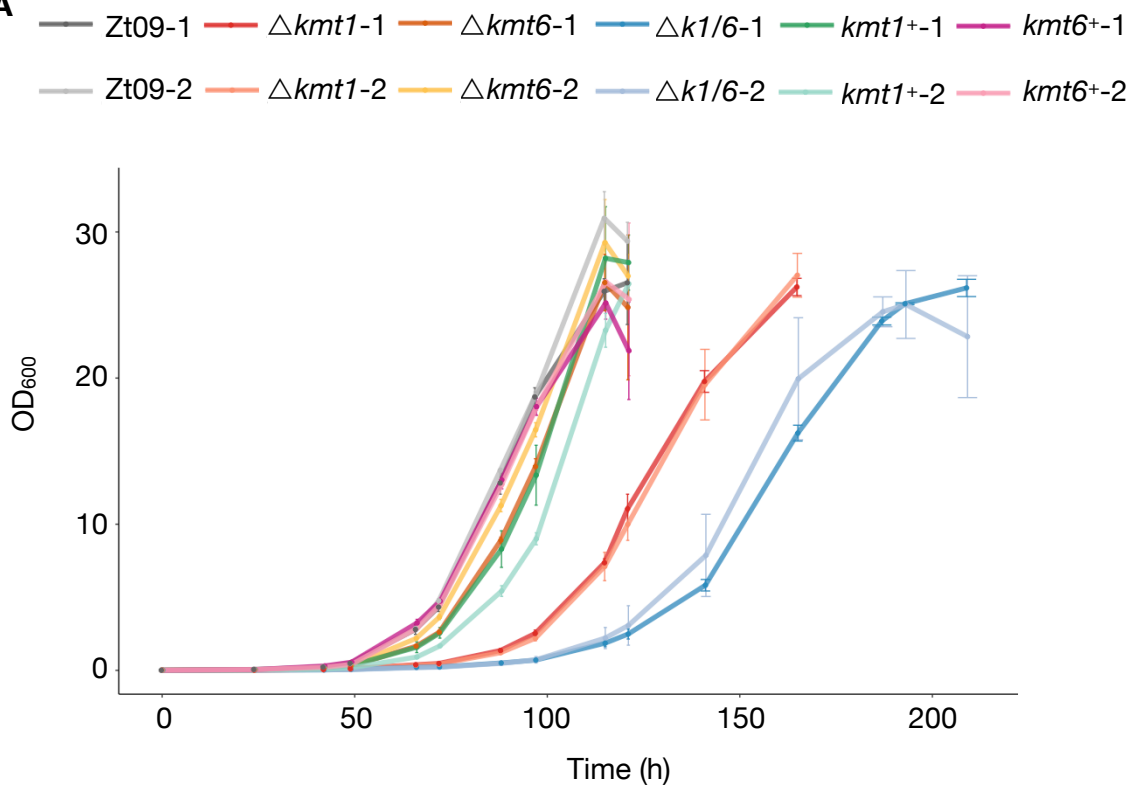**B**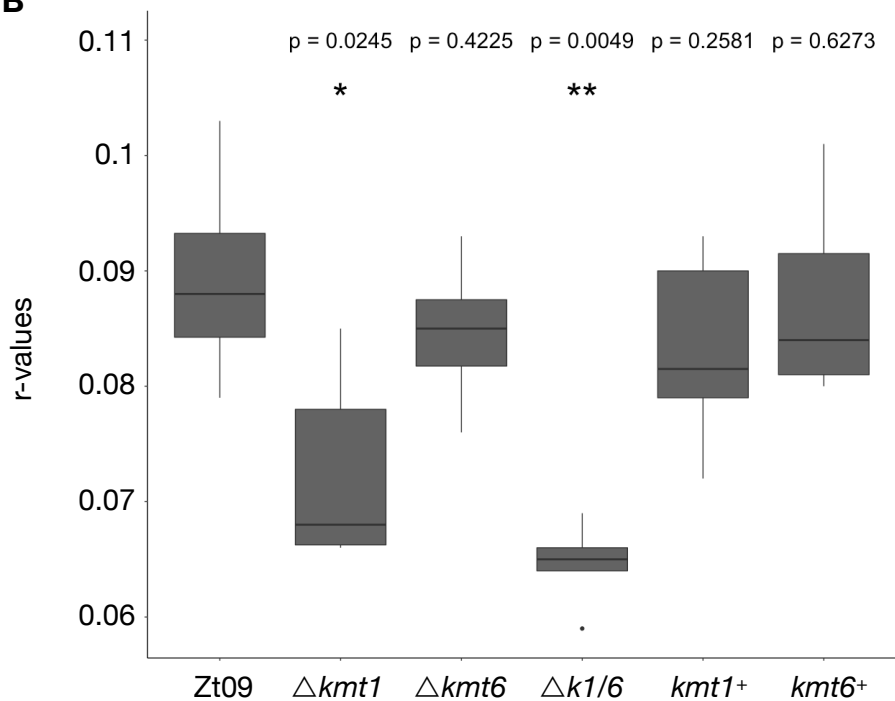

Supplement: S4 Fig — All strains were grown in liquid YMS medium at 18°C and the OD600 was measured until the stationary phase was reached (A). For each strain, two biological replicates were grown in technical triplicates each. The growth of Δkmt1 and Δk1/k6 mutants was impaired compared to Zt09 and Δkmt6 but was restored in complemented strains. (B) We used the R package growthcurver [89] to calculate r-values for each growth curve. The values for Δkmt1 and Δk1/k6 were significantly lower compared to Zt09, but this was not the case in the complemented strains and Δkmt6 (Wilcoxon-rank sum test, * p ≤ 0.05, ** p ≤ 0.01). (PDF) [file pgen.1008093.s017.pdf]

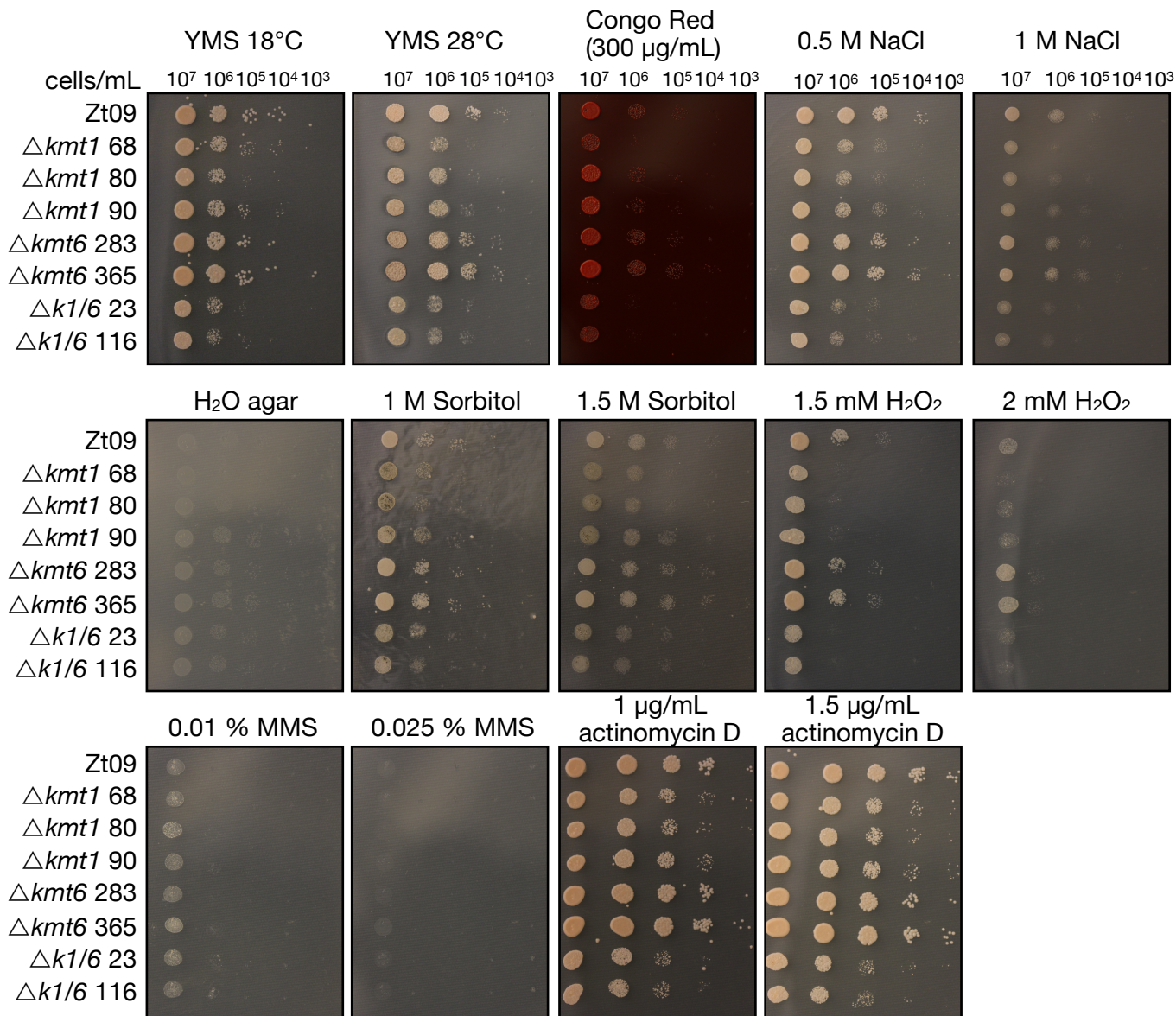

Supplement: S5 Fig — We observed almost no differences between Zt09 and Δkmt6 strains, whereas Δkmt1 and Δk1/k6 mutants displayed decreased growth, as observed in the growth rate comparison. (PDF) [file pgen.1008093.s018.pdf]

**A**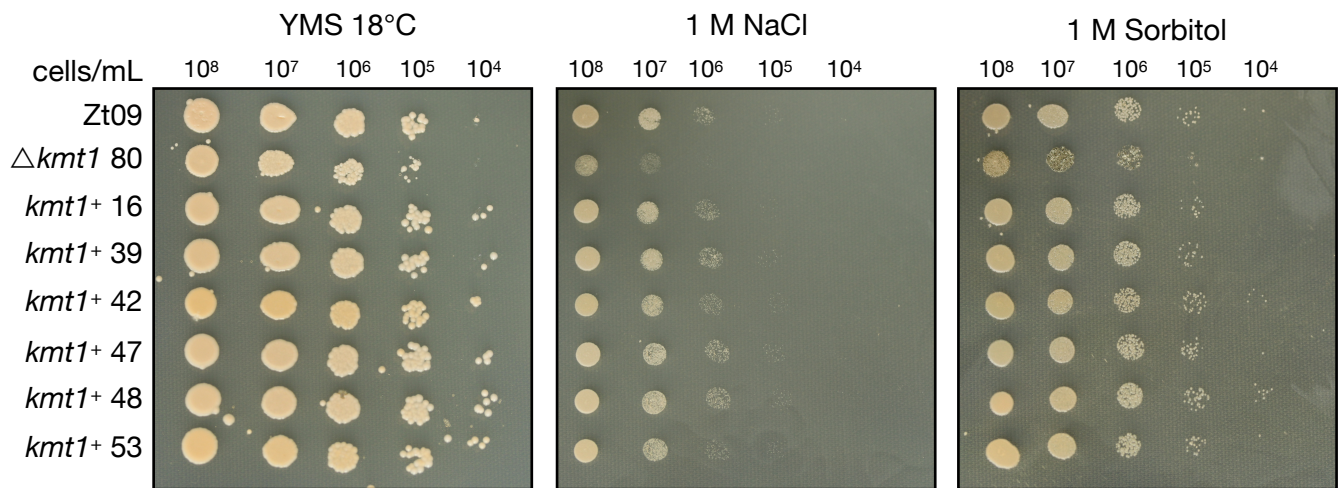**B**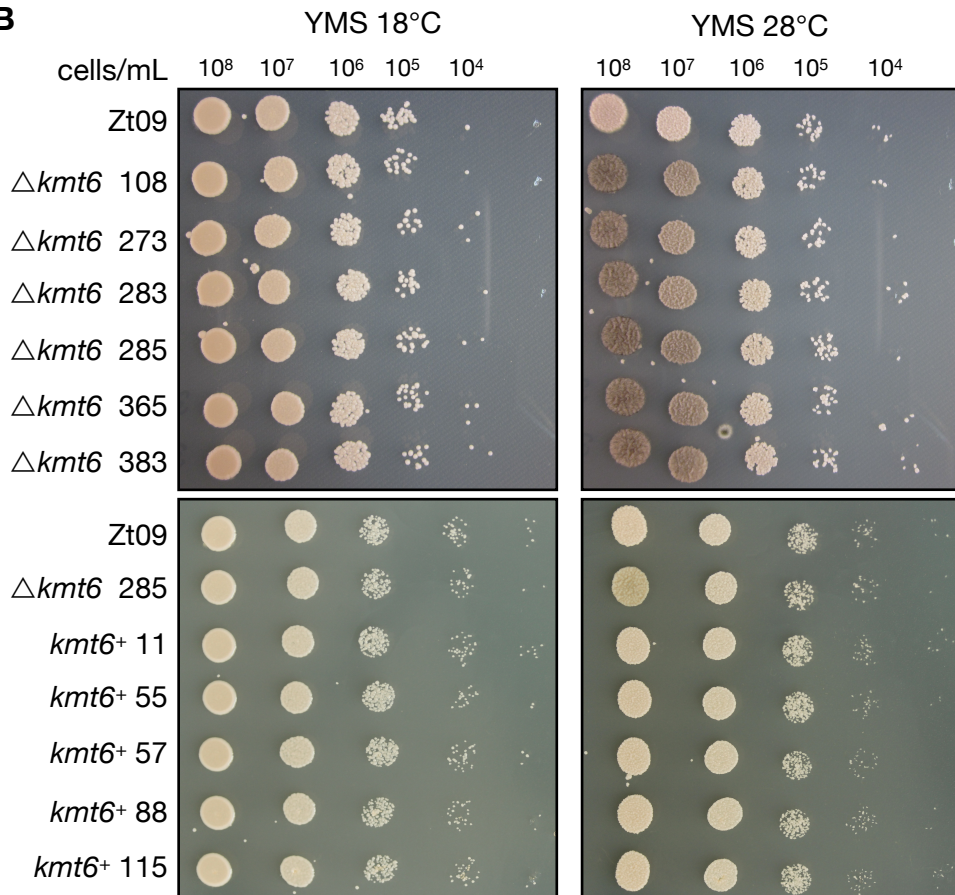

Supplement: S6 Fig — (A) The Δkmt1 mutants showed overall decreased growth and were particularly sensitive to osmotic stress. These phenotypes were restored in the complemented strains. (B) Increased melanization at high temperatures, observed in the Δkmt6 mutants, was also reversed in the respective complementation strains. (PDF) [file pgen.1008093.s019.pdf]

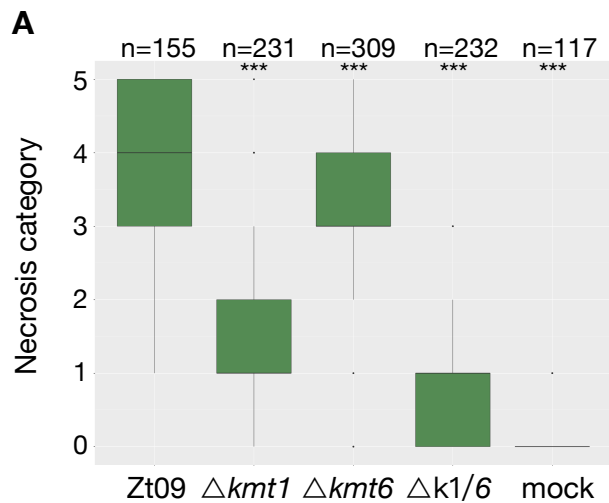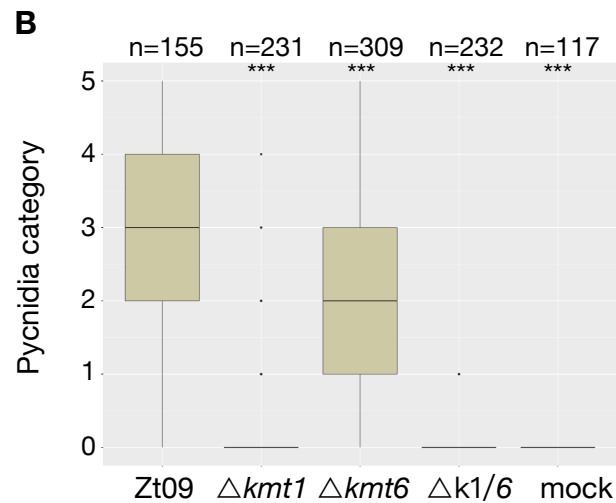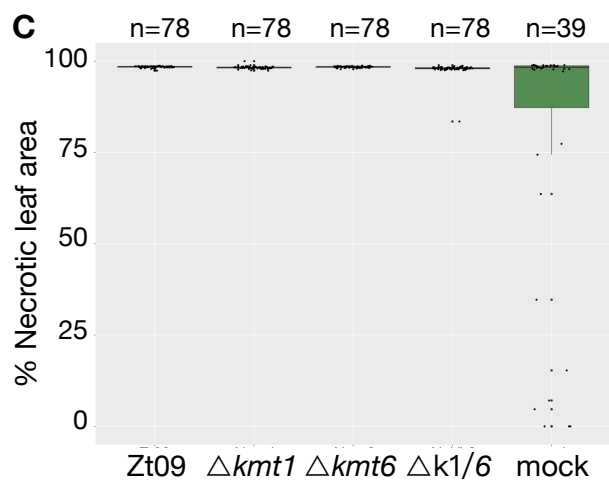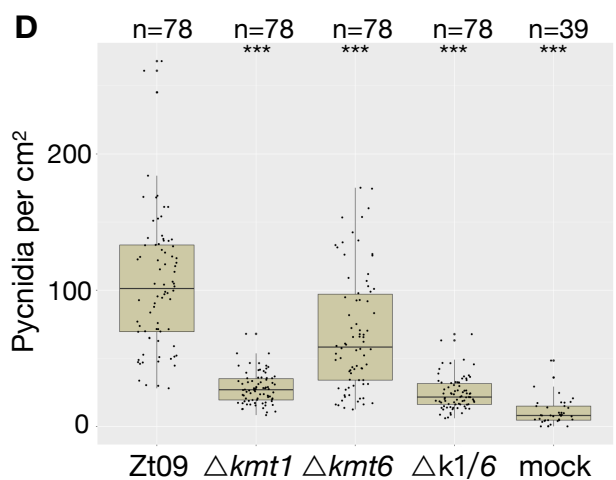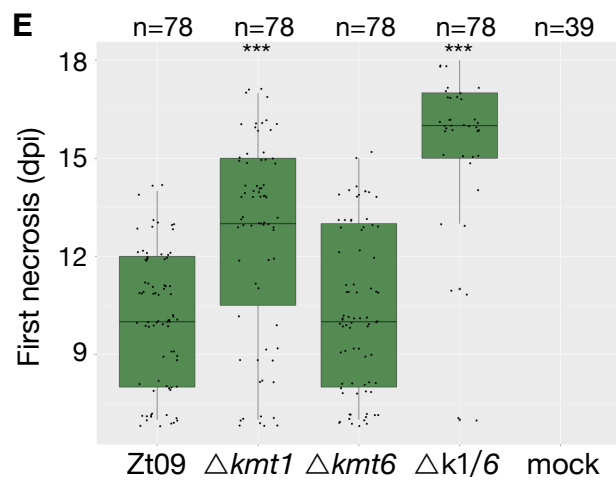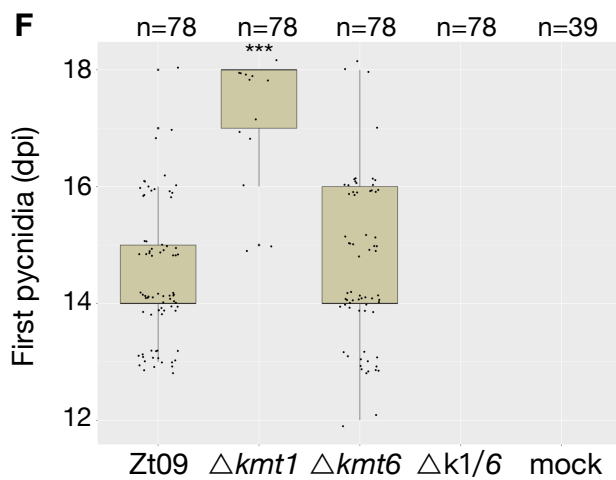

**G**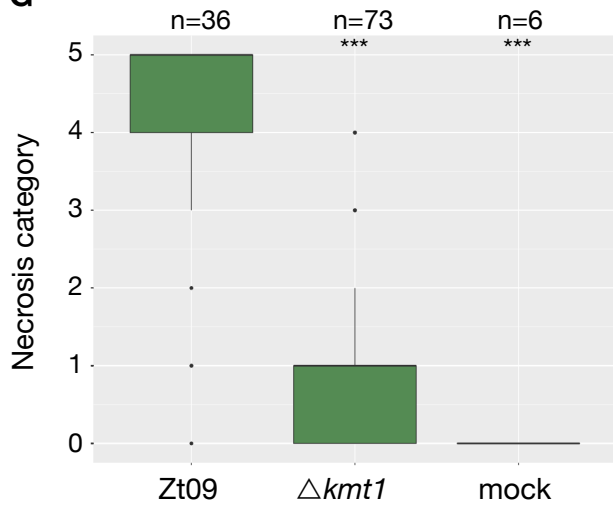**H**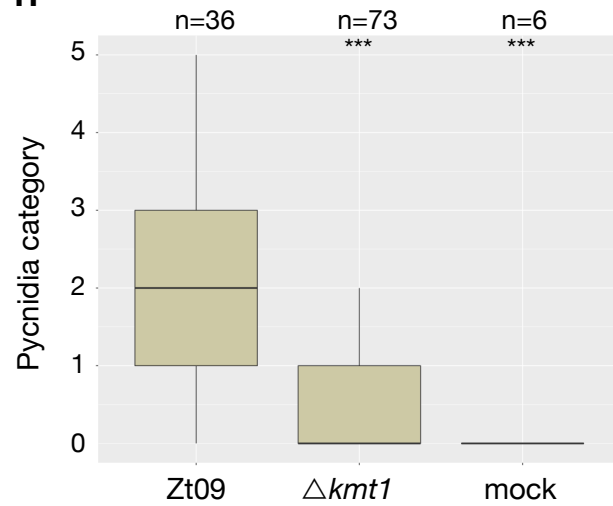

Supplement: S7 Fig — We conducted three independent experiments, including 40 leaves per treatment and using at least two biological replicates per strain. Infection symptoms were evaluated and compared as the percentage of leaf area covered with pycnidia (asexual fruiting bodies) and necrotic lesions within the inoculated leaf areas by manual inspection as well as by automated image analysis of scanned leaves [90]. Symptoms in form of necrotic lesions and pycnidia were quantified after 21 days of infection either manually (A) and (B) or by automated image analysis of infected leaves (C) and (D). Senescence on mock treated leaves was identified as necrosis by the automated image analysis and therefore all treatments, including mock treated leaves, show a high level of necrosis in this analysis. Furthermore, first appearance of symptoms was documented by daily screening of inoculated leaves (E) and (F). If no symptoms in form of necrosis or pycnidia appeared during the screening period, no data is shown for those treatments. Virulence of both, Δkmt1 and Δk1/k6 strains was highly impaired. Δkmt6 strains were still able to produce necrosis as well as pycnidia, but the symptoms were reduced compared to Zt09. To evaluate if the decreased symptoms in Δkmt1 strains were due to the observed growth defects in vitro, we prolonged the infection time for one additional week (G) and (H). Consistent with the results observed for 21 days post infection, the ability to infect was highly impaired and likely not correlated to the slower growth rate. Wilcoxon-rank sum test was performed to test for significant differences (* p ≤ 0.05, ** p ≤ 0.01, *** p ≤ 0.001). (PDF) [file pgen.1008093.s020.pdf]

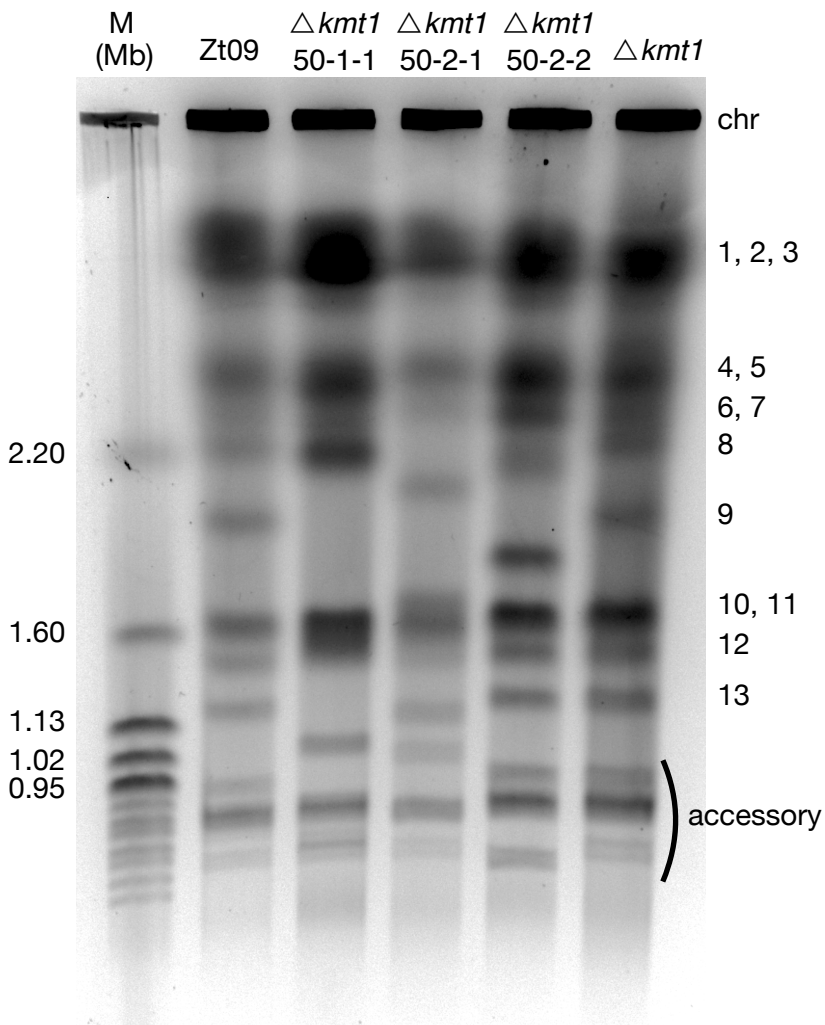

Supplement: S9 Fig — While there are no visible differences between the progenitor strains, all three single Δkmt1 clones exhibit different karyotypes. Chromosome size marker (M, in Mb) are Saccharomyces cerevisiae chromosomes (BioRad, Munich, Germany). (PDF) [file pgen.1008093.s022.pdf]

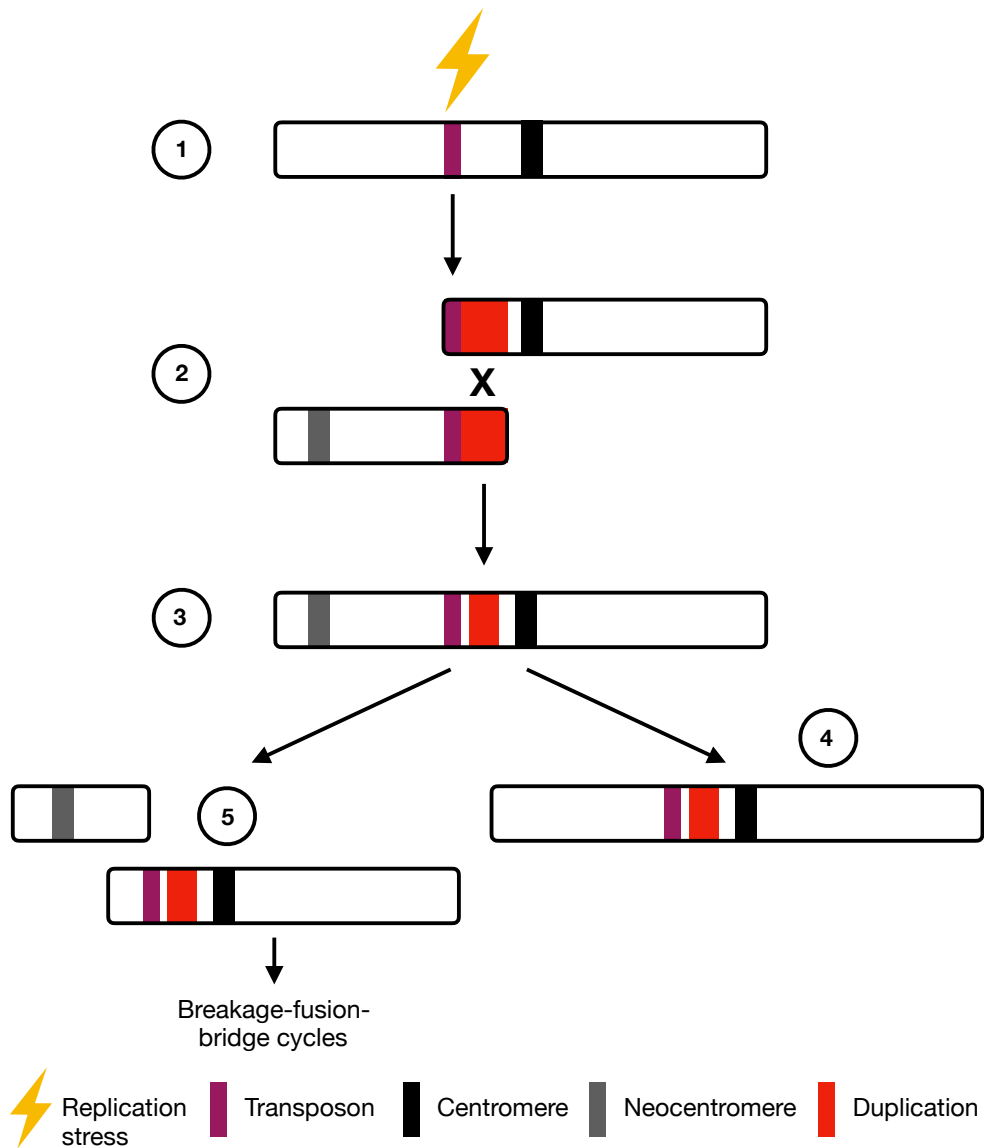

Supplement: S11 Fig — 1: Replication stress at repeated sequences enriched with relocalized H3K27me3 promotes structural variation in form of deletion or duplication. 2: Large segmental duplications arise during that process that are followed by chromosome breakage and new chromosomes are formed by adding de novo telomeric repeats at chromosomal breakpoints. While one of the chromosomal parts contains the original centromere, the other de novo chromosome forms a neocentromere. 3: The duplicated sequences are targets for mitotic recombination resulting in chromosome fusion. The chromosome is now dicentric. 4: To stabilize the chromosome, one of the two centromeres is inactivated, either epigenetically or by deletion of the underlying sequence. 5: Alternatively, the dicentric chromosome becomes unstable during mitosis and breaks between the two centromeres. The broken chromosome ends are repaired either by de novo telomere formation or fusion to a different chromosome, giving rise to new breakage-fusion-bridge cycles in following rounds of mitotic cell divisions. (PDF) [file pgen.1008093.s024.pdf]
