# Supplementary material for: Destabilization of chromosome structure by histone H3 lysine 27 methylation
Source: PLoS Genet. 2019 Apr 22;15(4):e1008093. doi: 10.1371/journal.pgen.1008093 (PMC6510446; doi:10.1371/journal.pgen.1008093)
Supplement: S8 Fig — (A) The short-term growth experiment over four weeks assessed stability of accessory chromosomes by screening individual clones in the populations for presence/absence of accessory chromosomes. Strains (Zt09, Δkmt1, Δkmt6, Δk1/k6) were grown in triplicates for four weeks and 4% of the population were transferred to fresh medium every three to four days. After four weeks, single clones were isolated and screened for the presence/absence of accessory chromosomes by PCR. (B) A long-term growth experiment over six months was conducted to monitor genome stability in Z. tritici populations. Three replicate populations per strain (Zt09, Δkmt1, Δkmt6) were grown in parallel exposed to the same growth conditions. 0.1% of the populations were transferred to fresh medium every three to four days. The genomes of the progenitor strains and all populations after six months of growth were sequenced to detect structural variation. (PDF) [file pgen.1008093.s021.pdf]

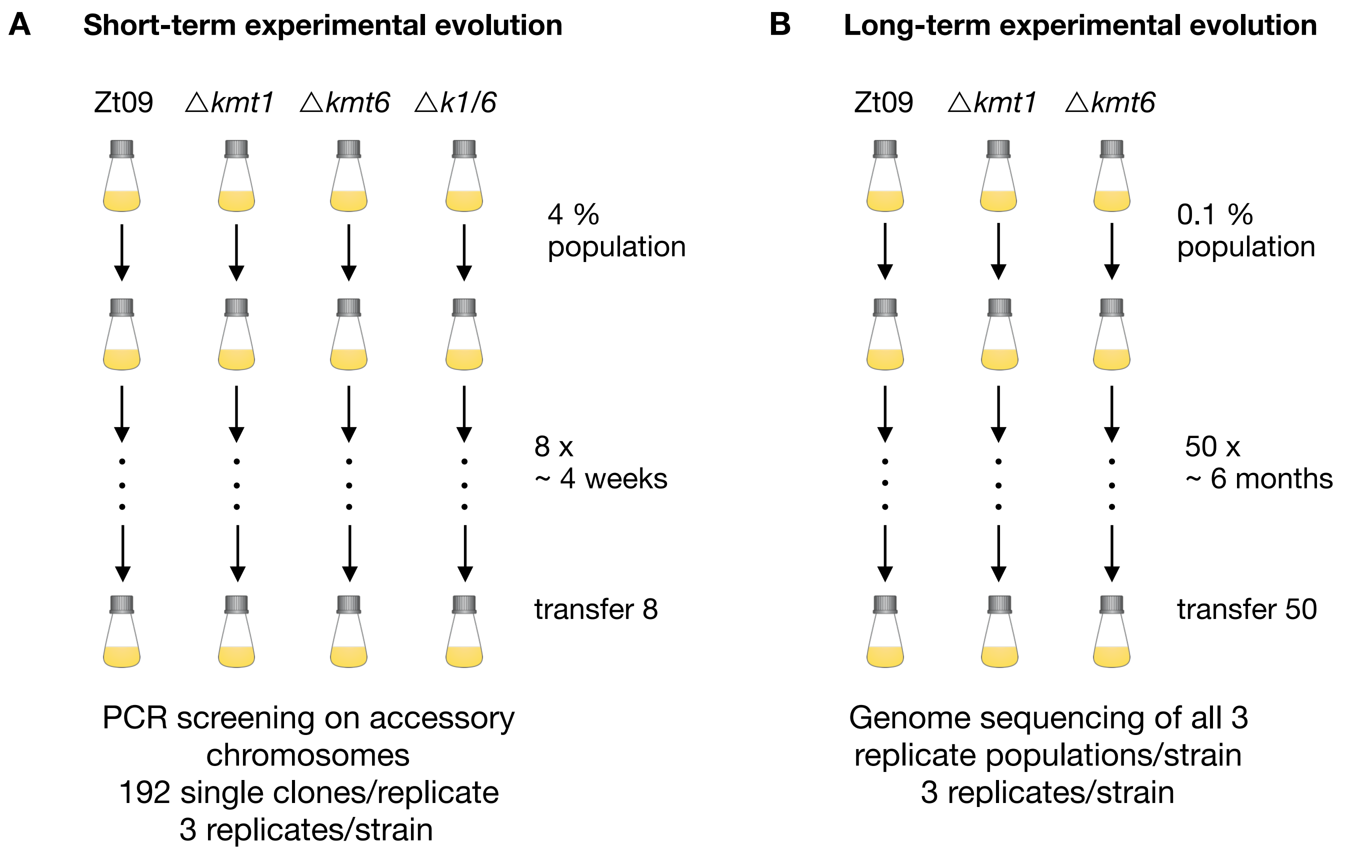
A
Short-term experimental evolution
Zt09
△kmt1
△kmt6
△k1/6
4 %
population
8 x
~ 4 weeks
transfer 8
PCR screening on accessory chromosomes
192 single clones/replicate
3 replicates/strain
B
Long-term experimental evolution
Zt09
△kmt1
△kmt6
0.1 %
population
50 x
~ 6 months
transfer 50
Genome sequencing of all 3 replicate populations/strain
3 replicates/strain
