# Supplementary material for: Destabilization of chromosome structure by histone H3 lysine 27 methylation
Source: PLoS Genet. 2019 Apr 22;15(4):e1008093. doi: 10.1371/journal.pgen.1008093 (PMC6510446; doi:10.1371/journal.pgen.1008093)
Supplement: S10 Fig — (A) Six duplicated regions were found in Δkmt1-50-1-1, two each on chromosomes 1, 9 and 13. The breakpoints of the first duplicated sequence on chromosome 1 fused to the right telomeres of chromosome 13 (1*13) and 19 (1*19), the second duplication is a tandem duplication. While the right telomere of chromosome 19 fused to chromosome 1, the left arm including the centromere is deleted and de novo telomere formation occurred at the breakpoint. Chromosome 13 has two duplicated regions, one is a tandem duplication and one shows de novo telomere formation on both ends indicating a breakage of chromosome 13. The right telomere is fused to chromosome 1, the first breakpoint of the first duplicated region provides the new left telomere of the 1*13 chromosome. This breakpoint is located very close to the centromere. The left arm including the centromere forms a new, smaller chromosome 13_1, ending at the right breakpoint of the first duplicated region with de novo telomeres. Chromosome 9 did not fuse with another chromosome, but the structural variation rather led to the formation of two smaller chromosomes, both containing the duplicated sequences. The larger chromosome 9 (9_1), ends at the first breakpoint of the first duplicated region with de novo telomeres and ends at the breakpoint of the second duplicated region with de novo telomeres. The second duplicated region ends at the end of the chromosome where de novo telomere formation occurred as a result of chromosome breakage (~12 kb). In the smaller chromosome 9 (9_2), the two duplicated regions fused, deleting the entire sequence between the duplicated regions. (B) In the clone Δkmt1-50-2-1, we detected four duplicated regions. While two are located on chromosome 9 and result in a very similar structural variation as described in (A), the other two are found on chromosome 1 and 8. One breakpoint of each duplicated region marks a fusion of the respective chromosomes, while the other one displays de novo telomere formati [file pgen.1008093.s023.pdf]

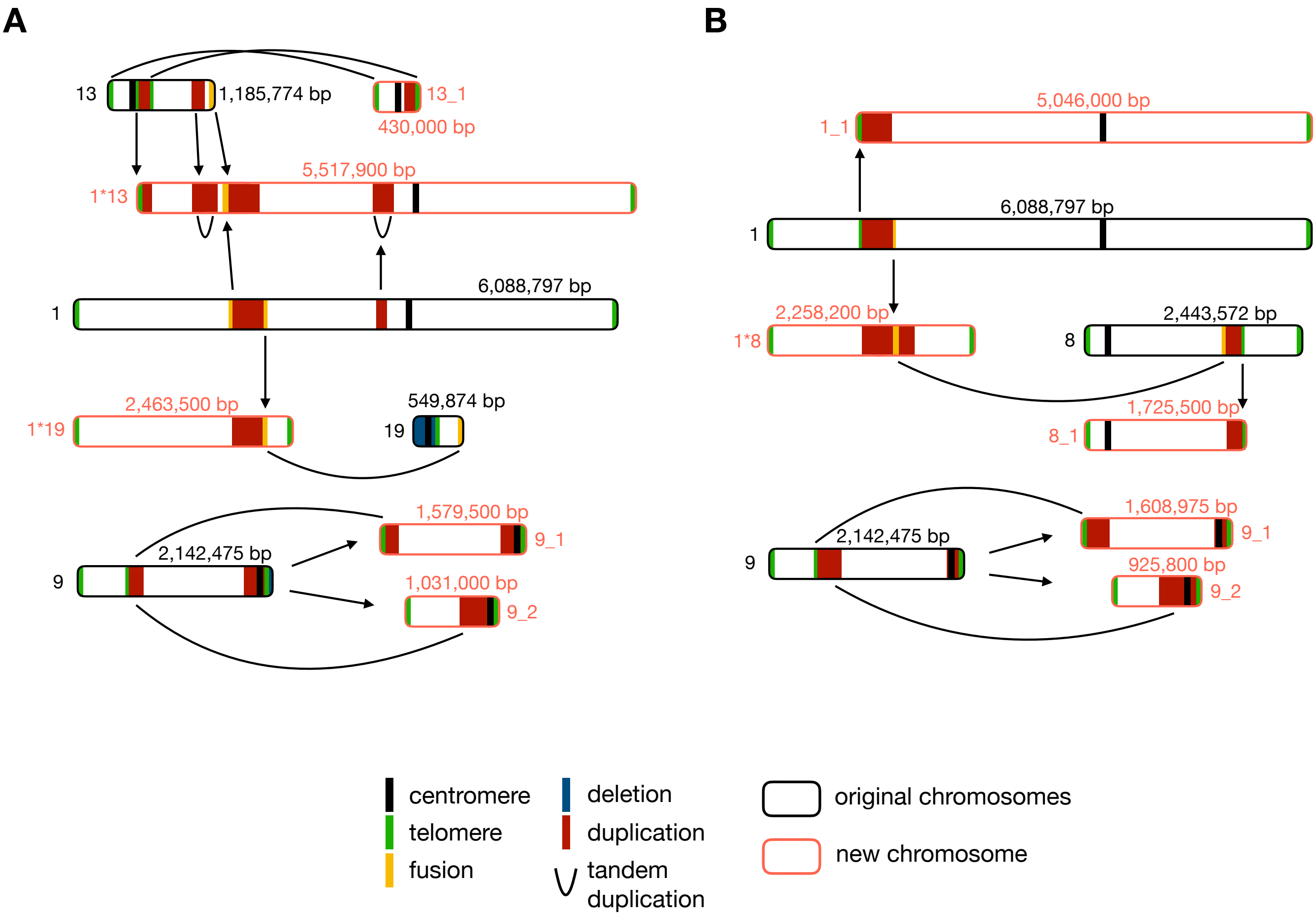
A
13
1,185,774 bp
13_1
430,000 bp
5,517,900 bp
1*13
1
6,088,797 bp
2,463,500 bp
1*19
549,874 bp
19
1,579,500 bp
9_1
2,142,475 bp
9
1,031,000 bp
9_2
B
5,046,000 bp
1_1
6,088,797 bp
1
2,258,200 bp
1*8
2,443,572 bp
8
1,725,500 bp
8_1
1,608,975 bp
9_1
2,142,475 bp
9
925,800 bp
9_2
centromere
telomere
fusion
deletion
duplication
tandem duplication
original chromosomes
new chromosome
